# Small area geographic variation in girl and boy child marriage in India: a district-level longitudinal analysis, 2016 and 2021

**DOI:** 10.7189/jogh.15.04248

**Published:** 2025-10-10

**Authors:** Jewel Gausman, Yun-Jung Eom, Rockli Kim, S V Subramanian

**Affiliations:** 1 Guttmacher Institute, New York, New York, USA; 2 Maternal and Child Health Nursing Department, School of Nursing, University of Jordan, Amman, Jordan; 3 Interdisciplinary Program in Precision Public Health, Department of Public Health Sciences, Graduate School of Korea University, Seoul, South Korea; 4 Division of Health Policy and Management, College of Health Science, Korea University, Seoul, South Korea; 5 Harvard Center for Population and Development Studies, Cambridge, Massachusetts, USA; 6 Department of Social and Behavioral Sciences, Harvard T. H. Chan School of Public Health, Boston, Massachusetts, USA

## Abstract

**Background:**

Sustainable Development Goal (SDG) target 5.3 aims to ‘eliminate all harmful practices, such as child, early, and forced marriage’. India is a signatory to the SDGs and tracks this indicator to measure national progress. Geographic inequity in the change in the district-level prevalence of child marriage over time in India has not been previously explored.

**Methods:**

We used data from the National Family Health Survey (NFHS) in India from 2016 (NFHS-4) and 2021 (NFHS-5). We aligned districts to be consistent over time. We included 720 districts for girls and 716 for boys. We used a four-level logistic regression model to partition the total variation of boy (<21 years) and girl child marriage (<18 years) in each year. We computed a precision-weighted prevalence of girl and boy child marriage, as well as within-district variation, using standard deviation measures.

**Results:**

Across districts, the average prevalence of child marriage decreased from 22.65% to 18.89% among girls and from 16.17% to 13.57% among boys. Inequality across districts in the prevalence of girl and boy child marriage decreased between 2016–21, and there was a negative association between district prevalence in 2016 and the change in district prevalence between 2016–21. There was a positive correlation between the difference in prevalence in boy (*r =* 0.72; *P* < 0.001) and girl (*r* = 0 · 60; *P* < 0.001) child marriage and the change in standard deviation across districts, indicating increasing inequality in the worst-performing districts.

**Conclusions:**

We provide new insight into recent progress and setbacks in eliminating child marriage across India. We found considerable district-level heterogeneity and clustering of poorly performing districts, coupled with increasing local inequality, especially within the worst-performing districts. Intervention and policy may be more effective if informed by local inequality.

Child marriage is a violation of human rights, and its elimination remains a global public health priority. Sustainable Development Goal (SDG) Target 5.3 aims to ‘eliminate all harmful practices, such as child, early, and forced marriage and female genital mutilation’ [1]. Failure to end child marriage is likely to hinder progress on at least nine other SDGs [2]. The interconnection between the SDGs highlights the broader implications of child marriage on global efforts to improve health, education, and gender equality. Ending child marriage issues not only protects the rights of children but also supports sustainable development and well-being for all. Global research, as well as that conducted in India, has highlighted that girls and young women married before the age of 18 years *vs.* those who are married later often have lower levels of contraceptive use, rapid repeated childbirths, and are more likely to suffer from gender-based violence [3,4]. There has been little research on the prevalence and health consequences of boy child marriage; however, limited existing research suggests that boy child marriage may be associated with increased undesired fertility [5].

Considerable progress has been made in reducing girl and boy child marriage in India over the last several decades, though declines in its prevalence remain uneven across states and union territories (UTs) [6]. Changes in the prevalence of child marriage over time, examined in aggregate at the state level, may mask variation among lower-level administrative units (*e.g.* districts). Studies have described extensive heterogeneity in the prevalence of girl child marriage at the sub-state level, as well as the clustering of high-prevalence districts across state lines [7]. Ecological differences between districts may help explain some of the observed heterogeneity across districts. Heavily impoverished districts and those with lower levels of education among women and girls tend to have a higher prevalence of girl child marriage [8]. On the other hand, interventions and policies designed to address child marriage are often driven at the district level, which may be more effective in reducing prevalence in some areas than others.

On an even smaller geographical scale, several studies have reported considerable area variation within districts in India across many health and development indicators [9–12]. These results highlight significant inequality across local geographic and administrative units (*e.g.* villages). Local-level inequality can inform interventions based on state- or district-level indicators, improving the identification and targeting of populations most in need.

Small-scale geographic inequality in the change in prevalence of child marriage over time across districts in India has not been previously explored. A recent study provided the first estimates of temporal changes in child marriage among boys and girls in India. The results suggest that while child marriage among girls and boys has declined at the national level between 1993–2021, subnational variation at the state level may reflect stagnation in the rate of decline across smaller geographic units, leading to uneven progress [6]. Furthermore, this study provided the first estimates of boy child marriage in India; however, the estimates provided aligned with the international definition (<18 years), rather than the Indian law (<21 years). As such, prevalence estimates of child marriage occurring among boys <21 years would provide a more contextually relevant understanding of the practice. A better understanding of the geographic heterogeneity in both girl and boy child marriage, in accordance with current Indian law at the sub-state level, could better support programmatic efforts and inform targeted policy at the appropriate administrative level. We describe the change in the prevalence of girl and boy child marriage between 2016–21 at the district-level in India. Additionally, we examine the geographic heterogeneity of girl and boy child marriage across and within districts at each period to explore the relationship between place-based inequality and performance.

## METHODS

### Data and sampling strategy

We used data from the National Family Health Survey (NFHS) in India from 2015–16 (NFHS-4) and 2019–21 (NFHS-5). For simplicity, we referenced only the final year of each survey. These surveys provide data on population health, nutrition, and well-being as part of the Demographic and Health Surveys Program [13]. Both surveys were designed to select clusters – villages in rural areas and census enumeration blocks in urban communities – with probability proportional to population size from districts within states, randomly choosing households through systematic random sampling within each cluster [14].

### Participants

We included women aged 20–24 years to align with the SDG indicator 5.3.1, and men aged 23–27 years, as the legal definition of child marriage for men in India is <21 years.

After restricting the study population by age criterion, we included 118 700 women aged 20–24 years nested within 29 023 clusters, 720 districts, and 36 states and UTs, and 14 054 men aged 23–27 years nested within 6973 clusters, 719 districts, and 36 states and UTs from the 2021 data set (**Table 1**). In the case of 2016 data set, we dropped several clusters (women n = 51; men n = 11) in the original data since they were unavailable in the updated geometry, resulting in 122 745 women aged 20–24 years nested within 27 752 clusters, 720 districts, and 36 states and UTs, and 16 327 men aged 23–27 years nested within 7712 clusters, 716 districts, and 36 states and UTs. We found no missing information on marital status and age at first cohabitation in either data set.

**Table 1:** Final analytical sample

	State	District	Cluster	Individual
**NFHS-4 (2016)**				
Women	36	720	27 752	122 745
Men	36	716	7712	16 327
**NFHS-5 (2021)**				
Women	36	720	29 023	118 700
Men	36	719	6973	14 504

### Definition of child marriage

We defined girl child marriage as women aged 20–24 years married before their 18th birthday to align with the definition of the SDG target 5.3.1. The current legal marriage age for girls in India is 18 years. For boy child marriage in India, the legal age of marriage differs from the global standard. The current legal marriage age for boys in India is 21 years. The Prohibition of Child Marriage Act, which set the legal age for marriage and provided for penalties for individuals involved in the perpetration of the practice, established these age limits in 2006 [15]. To measure child marriage in a way that is most policy-relevant in India, we use the Indian definition of child marriage, and thus define boy child marriage as men aged 23–27 years married before their 21st birthday. Women and men who had never married or were married at or after the legal age of marriage were assigned a value of 0, while those who married before the legal age were assigned a value of 1.

### District geometry

We sought to examine district-level variation in child marriage across India and how this variation changed over time between NFHS-4 and NFHS-5. Shifts in district borders and geographies have characterised India’s sub-national geography. In NFHS-4, there were 640 districts, and in NFHS-5, there were 707 districts. Therefore, we used an updated geometry of 720 districts, since the state of Andhra Pradesh (AP) created 13 new districts in April 2022 [16]. We included these new districts because none of the AP districts in NFHS-5 align with the state’s current district boundaries, making district-level interpretations for current policy meaningless.

To update the district boundaries for AP, we first linked Assembly Constituency (AC) boundaries with the 707 district boundaries provided by the Demographic and Health Surveys Program Spatial Data Repository [17]. The AP’s Chief Electoral Officer provided the necessary information to link the districts [16]. Representatives in each state legislature are elected from ACs, and the boundaries of these ACs are contained within the district boundaries for each state or UT. Therefore, the AC shapefiles in AP can be used to create updated district boundaries by merging AC polygons according to the linkage information from the AP’s Chief Electoral Officer, allowing us to create 720 districts for NFHS-5. The final step was adjusting the 720-district shapefile to have the same external boundary of India as per the Survey of India’s specifications [18].

To link clusters to the updated geometry, we kept the original primary sampling unit (PSU)-district linkages for the 694 unchanged districts in NFHS-5 and 577 unchanged districts in NFHS-4, using the linkages provided in the microdata. For the remaining 23 districts in AP for NFHS-5 and the 130 changed districts for NFHS-4, we assigned PSUs to the updated 720 district shapefile using a spatial join based on their Global Positioning System coordintes.

### Statistical analysis

We structured the final analytic sample into four levels: individual i (level 1), clusters j (level 2), districts k (level 3), and states l (level 4). Given this nested structure, we estimated a four-level model using a Markov Chain Monte Carlo (MCMC) procedure to determine the prevalence of child marriage in each district in India in 2016 and 2021. By maximising a likelihood function, MCMC procedures employ a Bayesian approach that uses prior knowledge [19]. We used a second-order predictive quasi-likelihood approximation with iterative generalised least squares to generate the priors [20]. We applied this approach to the model as logit (Pr_ijkl_) = β_0_ + (u_0jkl_ + v_0kl_ + f_0l_) for child marriage in each survey round, where β_0_ denotes the constant, and u_0jkl_ is the residual for clusters j, v_0kl_ for districts k, and f_0l_ for states l. We assumed each set of residuals to be normally distributed, with a mean of zero and variances of u_0jkl_ ~ N(0, σ^2^_u0_), v_0jk_ ~ N(0, σ^2^_v0_), and f_0ijk_ ~ N(0, σ^2^_f0_), where σ^2^_u0_ denotes within-district between-village/block variation, σ^2^_v0_ within-state, between-district variation, and σ^2^_f0_ between-state variation. Individual-level variance is not directly estimated for binary outcomes and is instead assumed to come from a logistic distribution with a fixed variance of π^2^/3 or 3.29 [21].

We used the estimated priors to run MCMC on the same model. We specified a burn-in of 500 cycles and monitoring of 5000 iterations of chains. We then used the MCMC residuals derived in this four-level model to calculate a precision-weighted prevalence of child marriage in each cluster as exp (β_0_ + (u_0jkl_ + v_0kl_ + f_0l_)) / (1 + exp (β_0_ + (u_0jkl_ + v_0kl_ + f_0l_)). Based on these estimates, we assessed within-district variability by computing the standard deviation for each district. Also, we calculated the mean prevalence for each district based on these precision-weighted estimates. Finally, we calculated the variance partitioning coefficient to assess the magnitude of variability across different levels as σ^2^_z_ / (σ^2^_u0_ + σ^2^_vo_ + σ^2^_f0_).

We used descriptive analyses and visual inspections to assess district-level patterns over time. We used box plots to evaluate the temporal changes in district-level inequalities. We assessed the magnitude of change in child marriage at the district level from 2016 to 2021 associated with the baseline district prevalence of child marriage using simple linear regression. We further examined whether a systematic pattern exists between district prevalence and within-district variability, calculating the correlation coefficient between the two measures. Finally, we created maps to show the geographic distribution of district prevalence of child marriage at each survey round, and how it changed from 2016 to 2021. For decile cutoffs to show district prevalence at each survey round, we used 2016 prevalence estimates to illustrate how each district’s child marriage prevalence changed over time. For cutoffs showing the change patterns of prevalence, we established four categories – extremely worsened, worsened, improved, and extremely improved – using the median increase and decrease values.

We used Stata, version 16 (StataCorp LLC, College Station, Texas, USA) for data management and *R*, version 4.2.2 (R Core Team, Vienna, Austria) for its advanced graphical capabilities. Additionally, we used MLwiN, version 3.05 runmlwin command [22] to produce the posterior estimates, as it is optimised to handle complex hierarchical data structures.

## RESULTS

Across districts, the average prevalence of child marriage decreased from 22.65% to 18.89% among girls and from 16.17% to 13.57% among boys. Inequality across districts in the prevalence of girl and boy child marriage decreased between 2016 and 2021 (Figure S1 in the **Online Supplementary Document**). For girl child marriage, the prevalence was 17.58% (interquartile range (IQR) = 12.76–30.34%) in 2016, and decreased to 15.59% (IQR = 9.90–25.49%) in 2021. For boy child marriage, the prevalence was 13.47% (IQR = 8.50–21.7%) in 2016, which decreased to 11.97% (IQR = 6.74–18.70%) in 2021. The district-level inequality in girl child marriage was about 30% higher than that in boy child marriage for both years.

Districts with a high prevalence of girl child marriage (>25.2%) were widespread across India in 2016 (Figure S1 in the **Online Supplementary Document**). Bihar (n = 37), Madhya Pradesh (n = 37), AP (n = 24), Assam (n = 24), and Rajasthan (n = 23) had the most districts with a high prevalence of girl child marriage in 2016. The states with the largest concentration of districts having a high prevalence of child marriage in 2016 were Tripura (100.0%), Bihar (97.4%), AP (92.3%), Jharkhand (91.7%), and West Bengal (90.0%). In 2021, the geographic distribution of districts with a high prevalence of girl child marriage mainly remained the same. However, notable reductions in the number of high-prevalence districts occurred in Northwestern and Central India. Furthermore, there was a reduction in the prevalence of child marriage in districts with a high prevalence of girl child marriage in 2016 that are adjacent to districts with a lower prevalence, with little change noted in districts surrounded by other districts with a high prevalence. In 2021, four of the five states with the most districts having a high prevalence of girl child marriage in 2016 remained the same in 2021: Bihar (n = 35), Assam (n = 20), West Bengal (n = 17), AP (n = 16), and Madhya Pradesh (n = 16). Absent from this list was Rajasthan, where considerable reductions in the district prevalence of child marriage occurred between 2016–21. In 2021, Rajasthan only had eight districts with a prevalence of girl child marriage >25.2%, which constitutes a 65.2% reduction. In terms of concentration, the percentage of districts with a high prevalence of girl child marriage remained at 100.0% in Tripura. Slight decreases in the concentration of high-prevalence districts were observed in Bihar (92.1%) and West Bengal (85.0%) when comparing 2016 to 2021, with dramatic reductions in the concentration of high-prevalence districts seen in Jharkhand (58.3%) and AP (61.5%). Between 2016 and 2021, Arunachal Pradesh also saw dramatic improvements, going from nearly half (45.0%) of districts having a high prevalence of girl child marriage to 0.0% in 2021.

**Figure 1:**
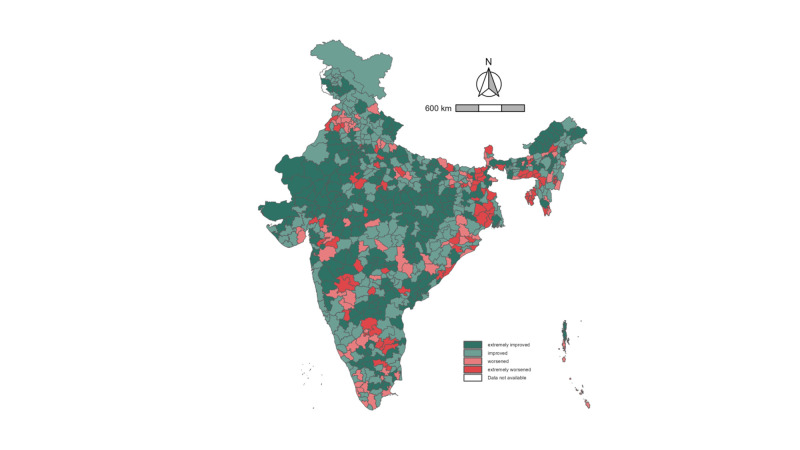
Map of change in prevalence of girl child marriage across districts in India, 2016 and 2021. *Cutoff values for four categories based on median increase and decrease values: extremely improved: −31.13 to −5.02%; improved: −5.01 to −0.01% (median: −5.02%); worsened: 0.01 to 2.26%; extremely worsened: 2.27 to 16.89% (median: 2.26%).

**Figure 2:**
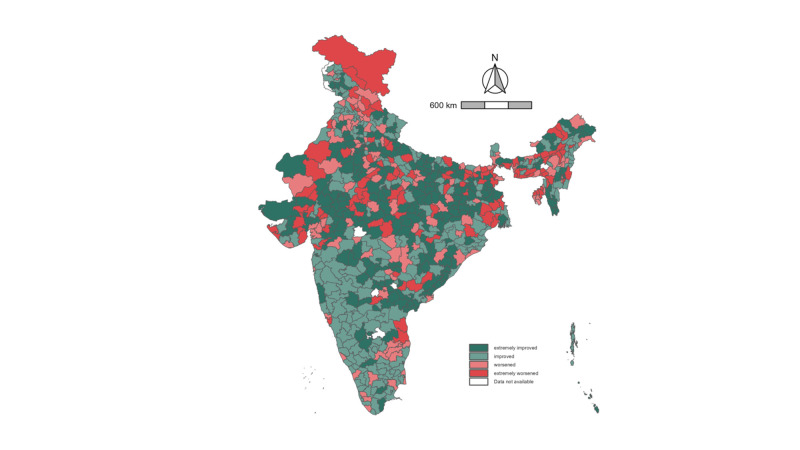
Map of change in prevalence of boy child marriage across districts in India. *Cutoff values for four categories based on median increase and decrease values: extremely improved: −33.05 to −4.22%; improved: −4.23 to −0.01% (median: −4.22%); worsened: 0.01 to 2.88%; extremely worsened: 2.89 to 16.37% (median: 2.88%).

Across India, 81 districts were categorised as being extremely worsened, with an increase in prevalence of girl child marriage of >2.27%, while 279 were extremely improved, which we defined as having a decrease of >5.01% in prevalence of girl child marriage between the study years (**Figure 1**). Districts with worsening trends were mainly in Southern and Northeastern India, whereas districts with a higher prevalence of girl child marriage were concentrated in Western and Central India. Rajasthan (93.8%) and Madhya Pradesh (91.7%) had the highest prevalence of districts categorised as extremely improved, whereas the Northeastern states of Tripura (71.4%) and Meghalaya (50.0%) had the highest prevalence of districts categorised as extremely worsened. A small cluster of districts stretching across Telangana (n = 2) and Arunachal Pradesh (n = 2) in Southern India was also categorised as extremely worsened over time (**Figure 2**). These districts tended to be concentrated in areas with a relatively low initial prevalence in 2016.

Geographically, districts with a high prevalence of boy child marriage (>16.7%) in both 2016 and 2021 were primarily concentrated in a band stretching across central India (Figure S3 in the **Online Supplementary Document**). There was relatively little change in the geographic patterning of districts with the highest prevalence of boy child marriage. Uttar Pradesh (n = 55), Madhya Pradesh (n = 46), Bihar (n = 31), Rajasthan (n = 31), and Gujarat (n = 23) had the most districts with a high prevalence of child marriage. The same states remained at the top of this list in 2021; however, Madhya Pradesh (n = 43) replaced Uttar Pradesh (n = 36) in the most districts with a high prevalence of boy child marriage. In 2016, Rajasthan (96.9%), Jharkhand (95.7%), and Madhya Pradesh (93.8%) had the highest prevalence of districts with a prevalence of boy child marriage >90%. In 2021, there was a reduction in district concentration in Jharkhand (78.3%) and Rajasthan (90.6%). Additionally, the concentration of high-prevalence districts increased in Bihar from 83.8% in 2016 to 91.9% in 2021.

In total, 245 districts across India were considered to have improved extremely over the study period, with a decrease in prevalence of boy child marriage of >4.22%, while 117 were considered to have worsened extremely, with an increase in prevalence of >2.88% (**Figure 2**). Several states saw dramatic improvement across districts, such as the National Capital Territory of Delhi, with 90.0% of districts having extremely improved, along with Mizoram (73.9%) and Jharkhand (65.3%). However, several states saw large percentages of districts worsen over time in the prevalence of boy child marriage. For example, Meghalaya had >80% of districts extremely worsened between 2016 and 2021 in the prevalence of boy child marriage. There was a reduction in extremes in the band stretching across Central India, where the prevalence of boy child marriage tended to be the highest, whereby districts with the highest prevalence appeared to improve and districts with the lowest prevalence tended to worsen (**Figure 2**, Figure S3 in the **Online Supplementary Document**). Furthermore, there was a worrying expansion of districts in which boy child marriage increased in Northern and Southern India, where overall prevalence tended to be lower in both 2016 and 2021.

**Figure 3:**
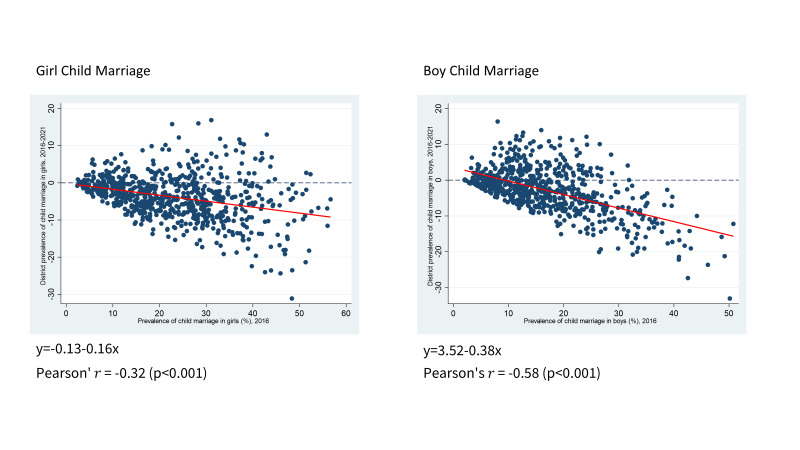
Correlation between baseline prevalence and change in prevalence in child marriage. **Panel A.** Girl child marriage. **Panel B.** Boy child marriage. *The horizontal axis is the 2016 district mean in child marriage. The vertical axis represents the change in district prevalence between 2016 and 2021. For girls, a total of 720 districts are included. For boys, a total of 715 districts are included since five districts (Khandwa (East Nimar), Sri Sathya Sai, Jangoan, Vikarabad, West Karbi Anglong) are excluded from analysis due to the unavailability of data for either of the two years.

Overall, there was a negative association between the baseline district prevalence of boy and girl child marriage in 2016 and the change in district prevalence between 2016 and 2021 (**Figure 3**). However, the strength of the association differs between girl and boy child marriage. There was a moderate negative correlation between the baseline mean district-level prevalence of girl child marriage and the change in district prevalence between 2016 and 2021 (*r* = −0.32; *P* < 0.001), while the correlation was stronger for boy child marriage (*r* = −0.58; *P* < 0.001).

While the variance partitioning estimates from the multilevel logistic regression models indicated that a substantial percentage of the variation in both boy and girl child marriage is attributable to differences between districts, small area variance at the village level constitutes a relatively larger proportion of the total variation observed in both girl and boy child marriage (Figure S4 in the **Online Supplementary Document**). For boy child marriage, the proportion of total variation at the district level decreased from 20.5% in 2016 to 16.1% in 2021, while it decreased from 23.3% to 19.9% for girl child marriage (Table S1 in the **Online Supplementary Document**). The proportion of total variation attributable to village level remained largely the same from 2016 (40.3%) to 2021 (40.2%) for boy child marriage. For girl child marriage, the proportion of total variance attributable to the village level decreased from 31.8% in 2016 to 27.2% in 2021. In both 2016 and 2021, there was a strong positive correlation between mean district prevalence and within-district variation for both boy and girl child marriage in 2016 and 2021 (Figure S5 and S6 in the **Online Supplementary Document**). For girl child marriage, there was a strong positive correlation between district prevalence and the district standard deviation of prevalence in 2016 (*r* = 0.87; *P* < 0.001) and 2021 (*r* = 0.93; *P* < 0.001). The strength of the correlation between district prevalence and standard deviation was similar for boy child marriage in 2016 (*r* = 0.86; *P* < 0.001) and 2021 (*r* = 0.91; *P* < 0.001). A strong, positive correlation was also observed between the difference in prevalence in boy (*r* = 0.72; *P* < 0.001) and girl (*r* = 0.60; *P* < 0.001) child marriage and the change in standard deviation across districts (Figure S7 in the **Online Supplementary Document**). These results indicate that within-district heterogeneity has increased in districts where child marriage prevalence has increased, but generally decreased in districts where it has decreased.

## DISCUSSION

We provide new insight into the geographic heterogeneity observed in recent progress and setbacks in eliminating child marriage across India. First, while aggregate estimates at the district-level point to considerable progress in reducing girl and boy child marriage between 2016–21, the reductions observed have not been uniform at the district level. Second, we find considerable district-level heterogeneity and clustering of poorly performing districts both within and across states for both girl and boy marriage. Third, our results point to increasing local inequality within districts in the change in prevalence of girl and boy child marriage over time, especially within the worst-performing districts.

Tracking progress at the sub-state level between 2016–21 is particularly important for policy and programmatic purposes, as it is a period during which states overall have experienced mixed and stagnating success [6]. For many health and development-related initiatives, policies and programmes are often targeted and monitored at the district level [23,24]. An example of this is the establishment of the Aspirational Districts Programme that was launched in 2016, which prioritised 112 districts for faster achievement of the SDG indicators [25]. District administration in India has considerable power to shape and guide policy [26]. Prior research has found that while aspirational districts were more likely than other districts to meet the SDG target for girl child marriage, only 108 districts in India were likely to meet the SDG target of eliminating girl child marriage by 2030 [26]. Other studies have shown a lack of effect of district-wide interventions to reduce child marriage in India, pointing to large exogenous changes in social and development indicators that may have confounded the intervention’s impact [27,28].

At the district level, we found that the prevalence of girl and boy child marriage has largely declined in recent years. Furthermore, inequality between districts has also improved for both girl and boy child marriage. Some states have been particularly effective in decreasing the prevalence of girl and boy child marriage across a large majority of districts, especially in Rajasthan for girl and the National Capital Territory of Delhi for boy child marriage. Interventions designed to prevent child marriage operating at the district level have been found to be effective in enabling context-specific adaptations that fostered a cascade effect to stimulate action at more hyperlocal administrative units, such as blocks and villages [29]. Other locally-driven interventions have successfully addressed the drivers of child marriage in India, but when applied in contexts such as Malawi, Mali, and Niger – where child marriage remains high – they have shown limited effectiveness, highlighting the need for context-specific interventions [30]. Furthermore, interventions often target areas where child marriage is already declining, overlooking areas where success has been limited [30]. Similarly, our findings suggest that progress has been muted in the worst-performing districts. We found that reductions in between-district inequality in boy child marriage have been greater than those observed in girl child marriage. In general, we would expect to see a stronger negative correlation across districts in the 2016 prevalence of girl child marriage *vs.* the change in prevalence between 2016–21 than was observed, given the high prevalence across many districts. The found correlation between baseline district prevalence and change in prevalence is weaker for girls than for boys may indicate that the drivers of child marriage are more deeply entrenched, or still poorly understood, in districts with widespread girl child marriage compared to that of boys. At the same time, there has been very little research on understanding the burden of boy child marriage in India. We add to the limited literature on boy child marriage in India, highlighting a substantial burden with considerable geographic heterogeneity.

We found that local inequality within districts is the highest among the worst-performing districts for both girl and boy child marriage. Furthermore, local-level inequality within districts with the highest prevalence of boy and girl child marriage appears to be growing over time. For both boys and girls, districts with increasing prevalence tend to have higher within-district variability, whereas districts with the largest reductions in prevalence show decreases in standard deviation. In these districts, district-wide intervention may not be sufficient to address the challenges faced by the poorest-performing villages that are driving the high rates of child marriage observed. These results are further supported by the large proportion of variation attributable to the village level we found. Targeting priority districts based on overall prevalence may mask the underlying small area variation, thus further increasing already existing inequities for populations most at risk. Moreover, policies and programmes targeting the worst-performing districts could worsen small-scale geographic inequalities if changes in within-district heterogeneity are not carefully considered.

Several limitations should be considered. First, while we used global indicators to measure the prevalence of girl child marriage, the indicator has faced some criticism [31]. The SDG indicator 5.3.1, which is overseen by the United Nations Children’s Fund, is widely interpreted as a measure of child marriage prevalence [32]. The United Nations Children’s Fund states that ‘The prevalence of child marriage is measured retrospectively among women whose risk of marrying in childhood is complete, *i.e.* those who are at least 18 years old, and the age group of 20–24 years is used by convention to represent the current prevalence of the practice’ [33]. Since the indicator is calculated retrospectively, it is essential not to confuse incidence with prevalence when interpreting its value. India uniquely defines boy child marriage as <21 years, whereas globally it is defined the same as girl child marriage (<18 years). Therefore, we created a new measure of boy child marriage to be consistent with the retrospective calculation of girl child marriage. We used the Indian legal definition to be consistent with Indian law, thus making our study most relevant for national policy in India and local stakeholders. Currently, there is legislation under discussion in India to increase the legal marriage age for girls to 21 years [34]. Furthermore, we must not discount the possibility of downward bias when asking individuals to report on their participation in an illegal practice. Data on age at marriage collected using the Demographic and Health Survey methodology (which includes the NFHS) are considered high-quality, and extensive analyses have examined potential biases in these estimates [35].

India, as a signatory to the SDGs, tracks this indicator to monitor progress in eliminating child marriage [36]. Our results support identifying geographic hotspots and priority areas for targeted interventions. Focusing interventions at the local level could improve efficiency in reducing the overall burden and achieve a more equitable impact.

## CONCLUSIONS

While India achieved overall reductions in child marriage between 2016–21, substantial district-level heterogeneity persists, with worst-performing districts experiencing increasing within-district inequality. The strong correlation between high district prevalence and local variation suggests that aggregate district-level interventions may be insufficient for areas with the greatest burden. Future research should examine village-level drivers of the persistent high prevalence of child marriage found in geographically clustered districts to identify intervention targets. Policymakers should consider complementing existing efforts with hyperlocal interventions targeting sub-district variation, particularly in high-burden areas where inequality is expanding. Incorporating village-level indicators in national monitoring systems could aid in identifying at highest risk and prevent masking of local disparities by district-level aggregation.

## Additional material


Online Supplementary Document
