# Supplementary material for: Small area geographic variation in girl and boy child marriage in India: a district-level longitudinal analysis, 2016 and 2021
Source: J Glob Health. 2025 Oct 10;15:04248. doi: 10.7189/jogh.15.04248 (PMC12512003; doi:10.7189/jogh.15.04248)
Supplement: Online Supplementary Document [file jogh-15-04248-s001.pdf]

Supplement to: Gausman J, Eom Y, Kim R, Subramanian SV. Small area geographic variation in girl and boy child marriage in India: a district-level longitudinal analysis, 2016 and 2021. J Glob Health. 2025;15:04248.

Small area geographic variation in girl and boy child marriage in India: a district-level longitudinal analysis, 2016 and 2021

Table S1: Exact variance estimates along with standard errors.

	Girl Child Marriage		Boy Child Marriage	
	2016	2021	2016	2021
	**Variance (SE)**			
State	0.509	0.706	0.521	0.521
District	0.264	0.266	0.272	0.191
Cluster	0.360	0.363	0.536	0.478
	**VPC (%)**			
State	44.9	52.9	39.2	43.8
District	23.3	19.9	20.5	16.1
Cluster	31.8	27.2	40.3	40.2

Figure S1: Box plots summarizing the distribution of child marriage across districts of India (2016 and 2021).

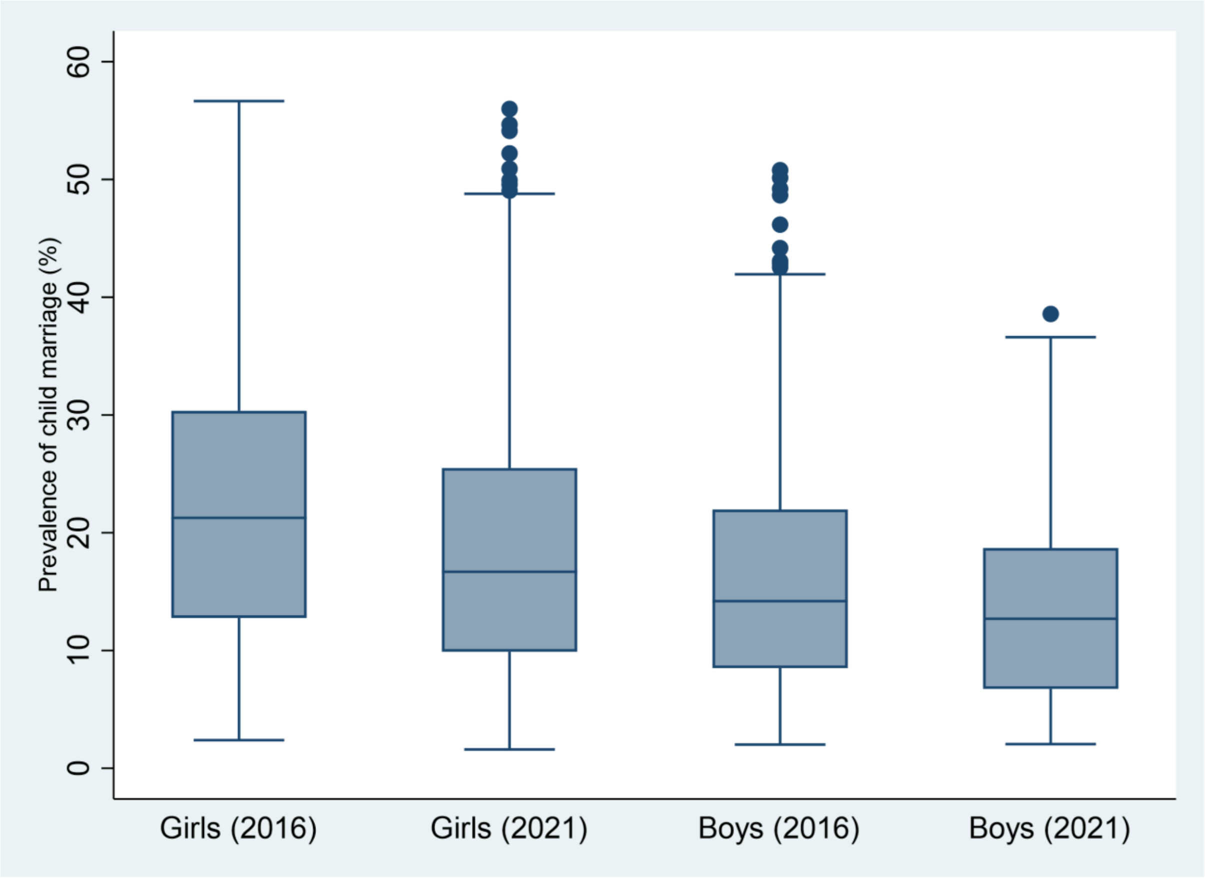

Figure S2. Map of the prevalence of girl child marriage across districts in India.

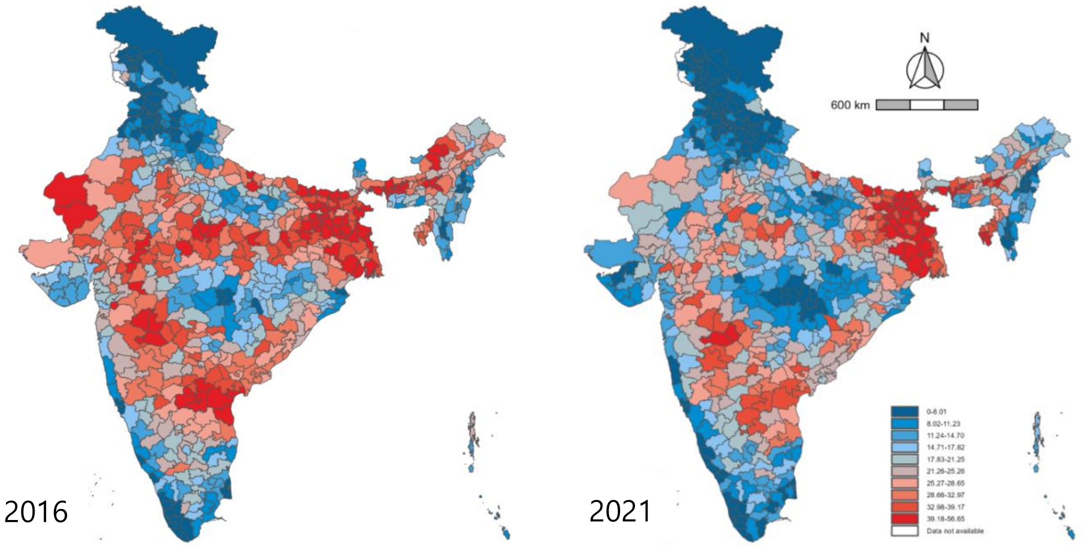

Note. Decile cutoff values based on mean prevalence in 2016. Girl child marriage data not available for two districts in 2016 and 2021 (Mirpur and Muzaffarabad in Jammu & Kashmir).

Figure S3. Map of the prevalence of boy child marriage across districts in India.

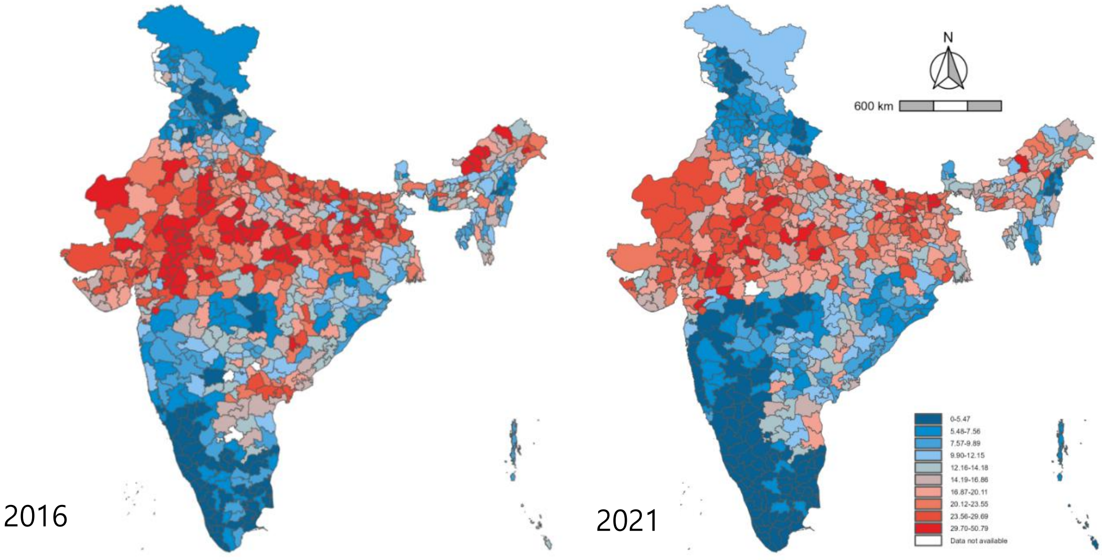

Note. Decile cutoff values based on mean prevalence in 2016. Boy child marriage data not available for 6 districts in 2016 (Mirpur and Muzaffarabad in Jammu & Kashmir, Sri Sathya Sai in Andhra Pradesh, West Karbi Anglong in Assam, and Jangoan and Vikarabad in Telangana.). Boy child marriage data not available for 3 districts in 2021 (Mirpur and Muzaffarabad in Jammu & Kashmir, Khanda (East Nimar) in Madhya Pradesh).

Figure S4. Proportion of total variance in girl and boy child marriage attributable to cluster, district, and state levels, 2016 and 2021.

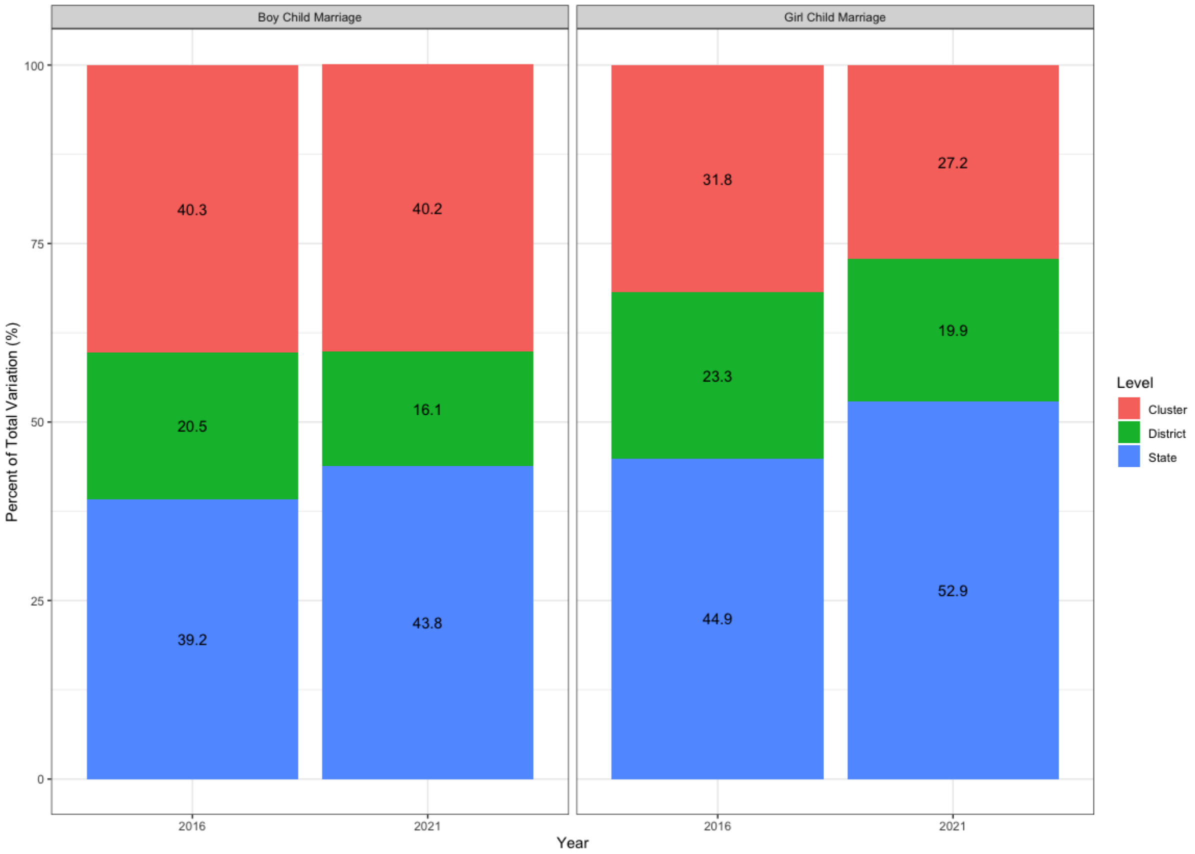

Figure S5. Correlation between mean prevalence and within-district variability in prevalence in girl child marriage.

2016 | 2021

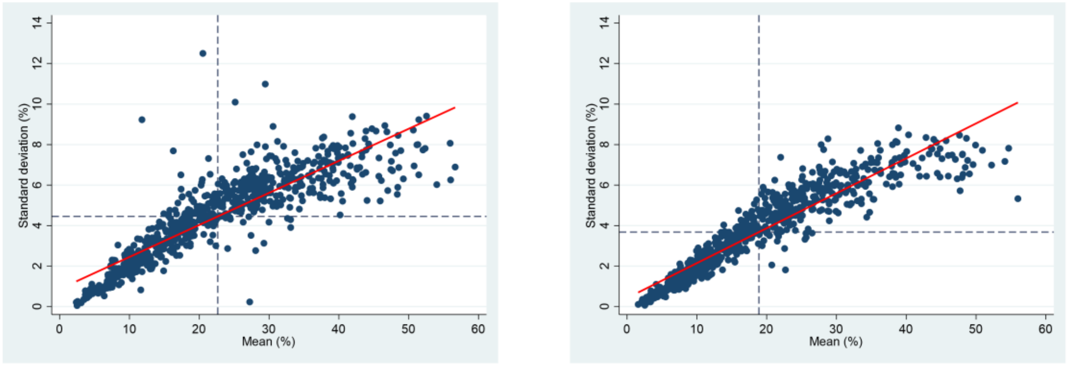

y=0.87+0.16x
Pearson' r = 0.87 (p<0.001)

y=0.42+0.17x
Pearson's r = 0.93 (p<0.001)

Note. 720 districts are included.

Figure S6. Correlation between mean prevalence and within-district variability in prevalence in boy child marriage.

2016

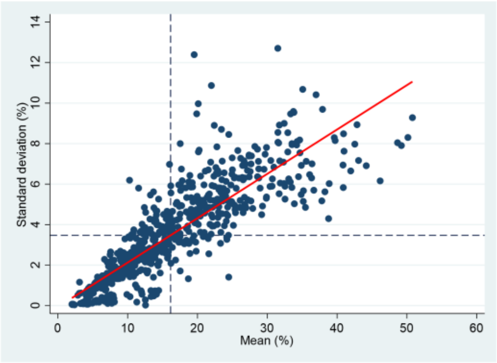

y=-0.07+0.22x

Pearson' r = 0.86 (p<0.001)

2021

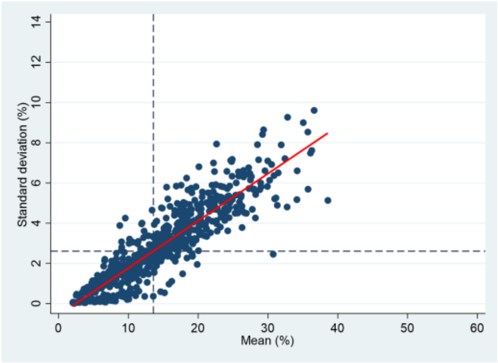

y=-0.58+0.23x

Pearson's r = 0.91 (p<0.001)

Note. In 2016, a total of 705 districts were included (Out of a total of 716 districts, 11 districts were excluded since they each had only one cluster). In 2021, a total of 717 districts were included (Out of a total of 719 districts, two districts were excluded since they each had only one cluster).

Figure S7. Correlation between change in prevalence and within-district variability in prevalence in child marriage.

Girl Child Marriage

Boy Child Marriage

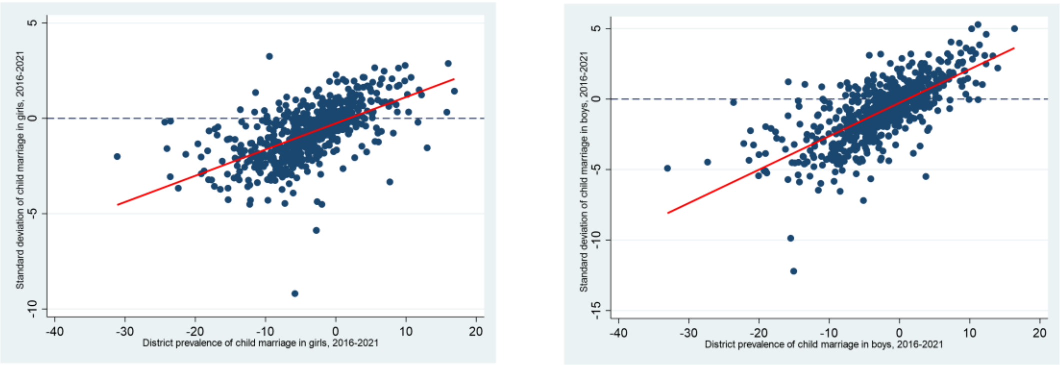

y=-0.26+0.14x

Pearson' r = 0.60 (p<0.001)

y=-0.26+0.24x

Pearson' r = 0.72 (p<0.001)

Note. Horizontal axis: mean change between 2016–21, vertical: standard deviation change between 2016–21. For girls, a total of 720 districts are included. For boys, a total of 703 districts are included, as five districts are excluded from analysis due to the unavailability of data for either of the two years, and 12 districts are excluded because each had only one cluster.
